# Ce, Gd and Yb accumulation in microalgae: an L-edge XAS study

**DOI:** 10.1107/S2053229625007156

**Published:** 2025-08-18

**Authors:** Maxence Plouviez, Karla Wolmarans, Benoit Guieysse, Andrea Marie E. Matinong, Olivia Buwalda, Valerie Mitchell, Pria Ramkissoon, Peter Kappen, Richard G. Haverkamp

**Affiliations:** a Cawthron Institute, 98 Halifax Street East Nelson 7010 New Zealand; b School of Food Technology and Natural Sciences, Massey University, Riddet Road, Palmerston North, 4410, New Zealand; c Australian Synchrotro, Australian Nuclear Science and Technology Organisation, 800 Blackburn Road, Melbourne, VIC, 3168, Australia; Oak Ridge National Laboratory, USA

**Keywords:** crystal structure, lanthanide, XAFS, EXAFS, Gd L_3_-edge, Ce L_3_-edge, Yb L_3_-edge, rare earth removal

## Abstract

The extraction and separation of rare earth elements (lanthanides) can be difficult due to their chemical similarities. Biological processes can have very selective activity towards different elements. We investigated the use of microalgae for this purpose by looking at the inter­action of Ce, Gd and Yb with the microalga *Chlamydomonas reinhardtii*, which has been induced to form polyphosphate granules. X-ray absorption spectroscopy with XANES and EXAFS at the Ce L_3_-edge, Gd L_3_-edge and Yb L_3_-edge was used to characterize the inter­action between the lanthanides and the phosphate-containing algae. All three of the lanthanides, added as the chloride salt to the algae, appeared to react to form phosphate com­pounds. These form both in the presence of phosphate granules or when there is only a low level of P present. CePO_4_ could be determined by XANES, but the structures of GdPO_4_ and YbPO_4_ were determined by EXAFS analysis without reference to the XAS spectra of the standard com­pounds. It is proposed that *C. reinhardtii* or other similar microalgae may be useful in the selective removal of rare earths from solution.

## Abstract 2

Ce, Gd and Yb can accumulate as lanthanide phosphate structures in the alga *Chlamydomonas reinhardtii* supplemented with phosphate and may be a means of selective recovery of rare earths from solution. EXAFS structural analysis of some Ce, Gd and Yb com­pounds are presented.

## Introduction

Rare earth elements (lanthanides) are important for many technological applications. These include phosphors for lighting and displays, batteries, magnets and catalysts (Hu *et al.*, 2024 ▸). Geopolitical considerations (Fan *et al.*, 2023 ▸) and the limited range of high-grade ore deposits available (Zhao *et al.*, 2023 ▸) provide incentives to find new sources of lanthanides that might include lower concentration deposits, by-product streams from other extraction processes and urban mining (Sedykh *et al.*, 2022 ▸). New extraction methods may be needed for the lower concentration sources, including radioactive waste sources (Jun *et al.*, 2023 ▸), and these methods could be greener, and more sustainable methods (Zapp *et al.*, 2022 ▸) include biomining (Vo *et al.*, 2023 ▸).

A possible method to extract lanthanides from solution could be adsorption or absorption by bacteria and algae (Birungi & Chirwa, 2014 ▸). For instance, the removal of La, Y, Sm, Nd and Eu from wastewater by bacteria was demonstrated by Sun *et al.* (2022 ▸) and Jacinto *et al.* (2018 ▸), and the removal of Y, Ce, Eu and Tb from solution by red algae has also been demonstrated (Iovinella *et al.*, 2022 ▸). Cyano­bacteria can absorb Ce from wastewater (Sadovsky *et al.*, 2016 ▸). It has also been shown that Cd can accumulate in the microalga *Chlamydomonas reinhardtii* (Penen *et al.*, 2017 ▸). These pieces of evidence suggest that biological methods could be a means to separate mixtures of lanthanides if the absorption is significantly different for different lanthanides (Plouviez *et al.*, 2024*b* ▸).

To enhance the absorption of lanthanides into algae, the presence of phosphate, including polyphosphate granules that can be generated in some microalgae (Plouviez *et al.*, 2024*b* ▸; Cliff *et al.*, 2023 ▸; Plouviez & Brown, 2024 ▸), has been considered (Plouviez *et al.*, 2024*b* ▸). This is based on the strong affinity that many rare earths have for phosphate, where the main mineral forms are monazite and xenotine, which are lanthanide phosphates. The affinity for phosphate increases along the lanthanide series from La to Lu (Wilharm *et al.*, 2021 ▸).

A suitable technique for studying the chemistry of lanthanides in the solid state is X-ray absorption spectroscopy, either the near-edge region with XANES (X-ray Absorption Near Edge Spectroscopy) to give chemical information or an examination of the crystal structure in the local environment of the element of inter­est using EXAFS (Extended X-ray Absorption Fine Structure). Both require similar data collection. For the rare earth elements, the X-ray absorption edges easily accessible include the K-edges and the L-edges. Specifically, the L_3_-edge has been shown to be more sensitive to changes in chemical environment (Asakura *et al.*, 2015 ▸, 2021 ▸).

It has been shown previously that Ce and Gd could absorb onto and within *C. reinhardtii* when the algae contains granules of polyphosphates (Plouviez *et al.*, 2024*b* ▸). Here we investigate the chemistry of Ce, Gd and Yb when this absorption has occurred, to understand the changes that have occurred in the rare earths with the P-containing algae. This range of Ce, Gd and Yb was chosen to represent a light, medium and heavy rare earth, with variation in the filling of electronic shells which should result in some slight differences in chemical bonding. Because standard phosphate com­pounds were not available for Gd and Yb, data measurements from an EXAFS analysis were required to assess whether these com­pounds had formed.

## Materials and methods

### Cultures

*Chlamydomonas reinhardtii* (CC-1690) culture main­ten­ance and cultivation were performed as described in Plouviez *et al.* (2021 ▸). Briefly, the microalga was sequentially cultivated on low-phospho­rus (1 mg P l^−1^) minimal media. The day of the experiment, 25 ml of culture was used to analyze the initial dry weight, optical density, total phospho­rus and dissolved phosphate, and also for microscopic observations (Plouviez *et al.*, 2021 ▸).

Algal cultures were supplemented with a P dose equivalent to a final concentration of 10 mg P l^−1^, using a 1 *M* stock solution of potassium phosphate (46 g l^−1^ KH_2_PO_4_ and 115 g l^−1^ K_2_HPO_4_) and either CeCl_3_, GdCl_3_ or YbCl_3_ at a final concentration of 6, 10 or 11 mg l^−1^, respectively.

The cultures were harvested by centrifugation at 10000 g for 3.5 min (Sigma 6–16 centrifuge) in several batches. The pellets were rinsed with distilled water and centrifuged again before being mixed to make a com­posite sample and frozen at −80 °C. The algae pellets were finally freeze dried in a Buchi Lyovapor L-300 for 36 h at 0.2 mbar (1 bar = 10^5^ Pa) with a tem­per­a­ture profile starting at −30 °C and finishing at 20 °C.

### XAS/XANES

The information provided here follows recent guidelines for reporting XAS data (Paripsa *et al.*, 2024 ▸). XAS scans at the Ce, Gd and Yb L_3_-edges were recorded at the MEX1 beamline at the Australian Synchrotron, ANSTO.

The MEX1 beamline uses X-rays from a bending magnet source and was configured for harmonic rejection for the energies of inter­est using a vertically collimating mirror translated to its rhodium stripe before a water-cooled Si(111) double crystal monochromator on an azimuth angle of 0°, and a vertical and horizontal focusing mirror also translated to its flat rhodium stripe after the monochromator. Energy resolution for MEX is (Δ*E*/*E*) of 1.4 × 10^−4^. Solid samples were pressed into 7 mm pellets for analysis. Standard com­pounds were mixed with cellulose powder and pressed into pellets for analysis and measured in transmission mode using 15 cm Ionitech gridded ion chambers filled with nitro­gen to an absolute pressure of 2 bar. Samples showing low concentrations (<2 wt%) of Ce, Gd and Yb were analysed in fluorescence mode using a four-element silicon drift detector. Samples were run using a beam size of 0.5 mm vertical × 3 mm horizontal, giving a photon flux of 3 × 10^11^ photons s^−1^. Ambient tem­per­a­ture (20 °C) and atmospheric pressure under helium were used as the sample environment. Beam energy was calibrated using Cr, Co and Cu metal foils for Ce, Gd and Yb, respectively, by aligning the peak of the first derivative of their respective K-edges to reference values.

Sample scans employed variable step sizes, with a large step for the pre-edge region, a very small step over the edge region, a larger step size for the post-edge (XANES) region, and a larger and variable step size increasing in proportion to k for the EXAFS region. The recorded energy range was expanded to include the edge energy of the reference metal foils with a small step size in the region of the reference metal edge. The energy range and step sizes used are listed in Table 1 ▸. This scanning was run in continuous scanning mode (not discrete steps).

*ATHENA* (Ravel & Newville, 2005 ▸) software was used to process the XAS spectra. *ATHENA* was used for background removal, with *E*_0_ unconstrained, and for spectra normalization.

### EXAFS

Data for EXAFS was recorded over a k of 10.6 Å^−1^ for Ce, 11.95 Å^−1^ for Gd and 11.8 Å^−1^ for Yb. Data processing and fitting used only a portion of this data range, as detailed in Table 2 ▸. Bond lengths were calculated using *ATOMS* and FEFF6 (within *ARTEMIS*) (Ravel & Newville, 2005 ▸), with CIF data taken from the Crystallographic Open Database (COD) (Gražulis *et al.*, 2009 ▸). *ARTEMIS* was used to fit spectroscopic data to crystal structure data. Where we had not been able to measure the spectra of the reference com­pounds of inter­est (GaPO_4_ and YbPO_4_), we calculated the EXAFS spectra from the CIF data taken from the COD (Table 2 ▸), and these are also available on the Materials Project, where a com­pilation of many FEFF files are also available (Jain *et al.*, 2013 ▸; Mathew *et al.*, 2018 ▸).

### Inductively coupled plasma mass spectrometry (ICP–MS)

The samples were prepared by the following method. The sample (1.0 ml) was added to a 15 ml centrifuge tube and 1.5 ml 69% HNO_3_ (Tracepur, Merck) and 0.5 ml 37% HCl (Tracepur, Merck) were added. The vessels were sealed and placed in a 100 °C water bath for 30 min. The digest was diluted with 13 ml Type-1 water to a 15 ml final volume. The solutions were qu­anti­tatively analysed for desired elements on an Agilent 7700 ICP–MS in He mode to reduce polyatomic inter­ferences. Calibration standards were prepared in a matrix matched solution from 1000 ppm single element standards (CPI Inter­national, USA). A 20 ppb solution of Y was used to monitor drift and matrix effects. All results are in µg g^−1^ and have been back calculated to the original sample.

## Results

### P, Ce, Gd and Yb accumulation in *C. reinhardtii* samples

The transition from P deplete to P replete conditions in the absence of added rare earths triggered P accumulation in *C. reinhardtii* up to (2.50 ± 0.42)% P in the dry weight of algae. Similar concentrations were measured for the cultures supplemented with P and the rare earths with (2.40 ± 0.31)% P, (2.60 ± 0.01)% P and (2.32 ± 0.15)% P for the samples supplemented with Ce, Gd and Yb, respectively. ICP–MS analysis of dried *C. reinhardtii* samples showed that the microalgae supplemented by both P and either Ce or Gd accumulated Ce at 0.57 ± 0.54% Ce and Gd at 1.5 ± 0.9% Gd. This absorption amounts to 17% of the Ce and 27% of the Gd available from solution.

**Figure 1:**
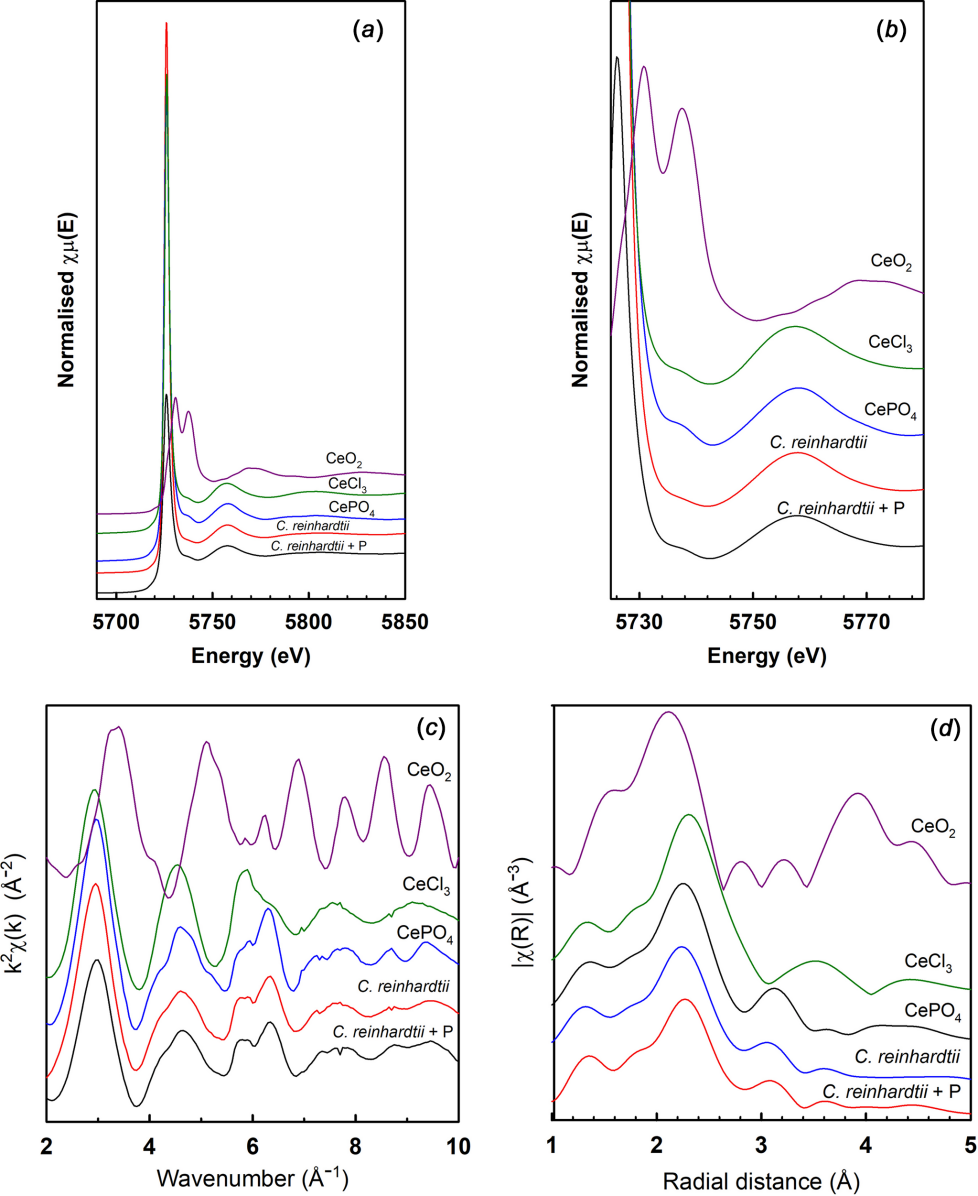
Ce L_3_-edge XAS for *C. reinhardtii* and standard com­pounds. (*a*) XANES, (*b*) near-edge region, (*c*) EXAFS function k^2^χ(k) and (*d*) Fourier transform moduli of the EXAFS spectra.

A large shift (3.4 eV) is observed in *E*_0_ (the absorption edge energy) in the XANES spectra with oxidation state, with Ce^IV^O_2_ at 5729.0 eV and Ce^III^Cl_3_ and Ce^III^PO_4_ at 5725.3 and 5725.5 eV, respectively. With algae, the oxidation state of Ce, based on *E*_0_, is clearly III at 5724.6 eV with higher P level and 5724.9 eV with only low levels of P, which is not a significant shift. In the near-edge region [Fig. 1 ▸(*b*)], the difference between the algae with or without extra P and between CeCl_3_ and CePO_4_ are subtle and not helpful in identifying differences in structure. In the k plots [Fig. 1 ▸(*c*)], the structure of CeCl_3_ is clearly differentiated. It is then apparent that the *C. reinhardtii* samples do not contain CeCl_3_, but com­pounds much closer to CePO_4_. This is also supported by the R plot [Fig. 1 ▸(*d*)], where the different bond lengths of CeCl_3_ than CePO_4_ are apparent.

### XAS Ce

The bond lengths determined from an EXAFS analysis shown in Fig. 1 ▸(*d*) clearly differentiate between the different reference com­pounds and identify that CePO_4_ is the dominant form of Ce in the algae. The most intense scattering peaks determined from FEFF6 calculations from published structures for these com­pounds are given in Table 3 ▸. The shortening of the Ce—O bond at higher oxidation state is clearly visible in the data recorded here. The distinctive Ce—P bonds at 3.2–3.3 Å are a clear characteristic of CePO_4_ which is visible in the R plots.

From the bond lengths it is apparent that the *C. reinhardtii* samples do not contain CeCl_3_, but rather contain CePO_4_, corroborating the XANES *E*_0_ data discussed above. There appear to be no difference in the structure of the Ce com­pounds in *C. reinhardtii* with or without P. Clearly, CePO_4_ forms with the algae in both cases, and that there is enough P present for this under P-limited conditions for normal cell growth. No significant portion of Ce remains as CeCl_3_, the form in which it was added.

### XAS Gd

For the Gd analysis, no Gd phosphate standard was available for com­parison. We therefore relied on EXAFS analysis of the bond lengths, and a com­parison with EXAFS calculated from available crystal structure data, for a full inter­pretation. EXAFS analyses of GdPO_4_ measured at the L_3_-edge have been reported previously (George *et al.*, 2010 ▸; Morss *et al.*, 1996 ▸; Yoon *et al.*, 2002 ▸).

**Figure 2:**
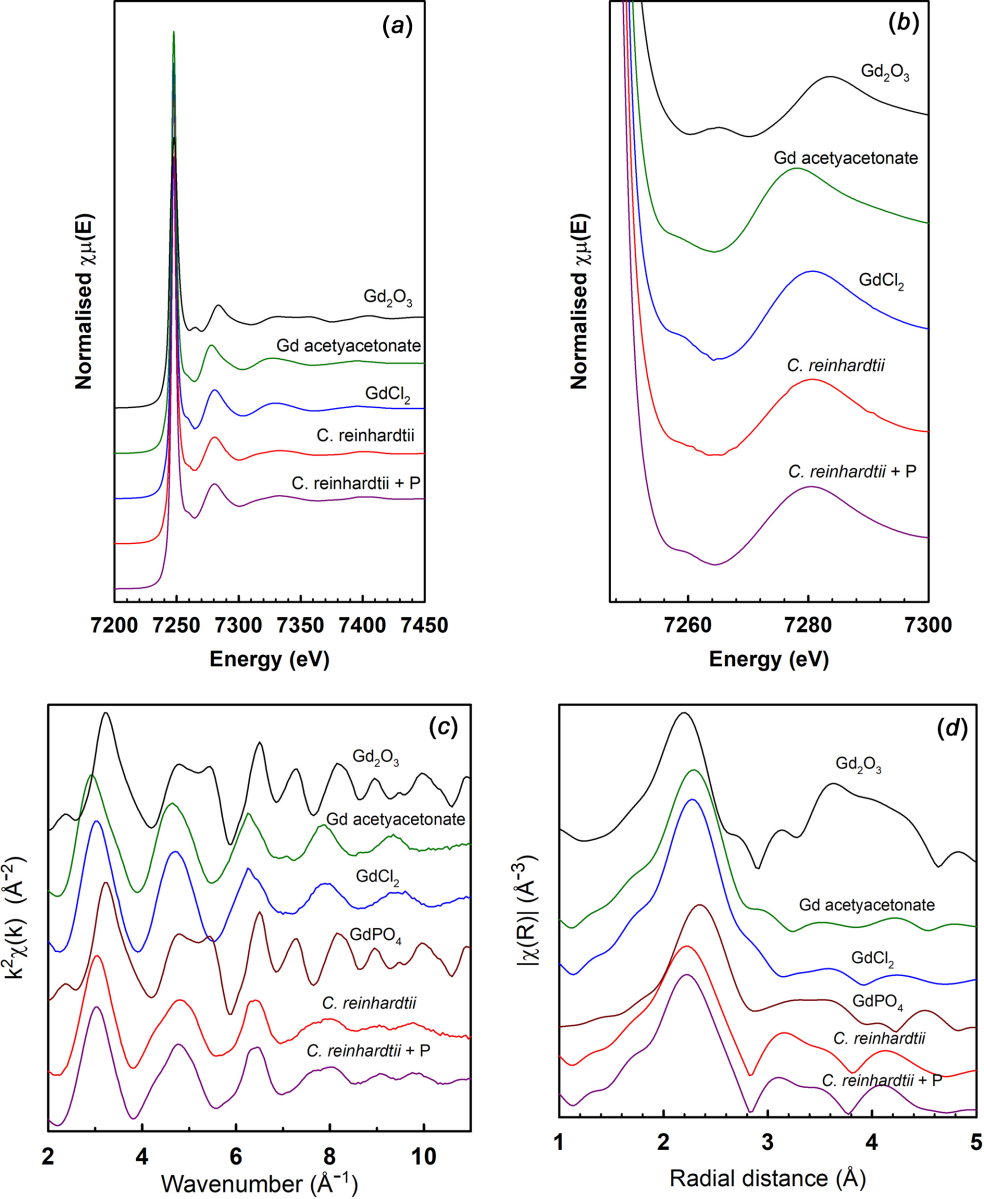
Gd L_3_-edge XAS for *C. reinhardtii* and standard com­pounds. (*a*) XANES, (*b*) near-edge region, (*c*) EXAFS function k^2^χ(k) and (*d*) Fourier transform moduli of the EXAFS spectra. GdPO_4_ in parts (*c*) and (*d*) are derived spectra from literature crystal structures.

In the near-edge region, the difference between the algae with or without extra P and between GdCl_3_ and Gd acetyl­ace­ton­ate are subtle and not helpful in identifying differences in structure, although Gd_2_O_3_ is clearly different and not similar to the algae samples or the other standards. In the near-edge region, the Gd in *C. reinhardtii* looks the same with and without extra P (Fig. 2 ▸).

However, in the k plot, there are clear differences between Gd in *C. reinhardtii* and the standards GdCl_3_ and Gd acetyl­ace­to­nate in the range 8.5–10.5 Å^−1^. The differences are also pronounced in the R plot, where the peaks at 3.2 and 4.1 Å in the algae samples are not present in the standards (Fig. 2 ▸).

The bond lengths determined from an EXAFS analysis clearly differentiate between the different reference com­pounds and identify that GdPO_4_ is the dominant form of Gd in the algae. The most intense scattering peaks determined from FEFF6 calculations from published structures for these com­pounds are given in Table 4 ▸. The bond lengths of 3.2 and 4.1 Å corresponding to Gd—P and Gd—Gd provide strong evidence of the presence of GdPO_4_ in the algae samples, both with extra P and with low levels of P. To confirm this, a full fitting of the crystal structure of GdPO_4_ to the EXAFS data for the *C. reinhardtii* with P granules was performed (using *ARTEMIS* running FEFF6) (Fig. 2 ▸). This provided an adequate fit to the structure.

### XAS Yb

For the Yb analysis, we also did not have a Yb phosphate standard for com­parison. We therefore again relied on EXAFS analysis of the bond lengths, and a com­parison with EXAFS calculated from available crystal structure data, for a full inter­pretation. An EXAFS analysis of YbPO_4_ measured at the L_3_-edge has been reported previously (Louvel *et al.*, 2015 ▸), as well as a Raman spectroscopy analysis (Becker *et al.*, 1992 ▸).

In the near-edge region, the difference between the algae with or without extra P and between YbCl_3_ and Yb in *C. reinhardtii* are subtle but, based on the experience with Ce and Gd, we do not expect this to be definitive in identifying differences in structure, although Yb_2_O_3_ is clearly different and not similar to the algae samples or YbCl_3_. In the near-edge region, the Yb in *C. reinhardtii* looks the same with and without extra P.

**Figure 3:**
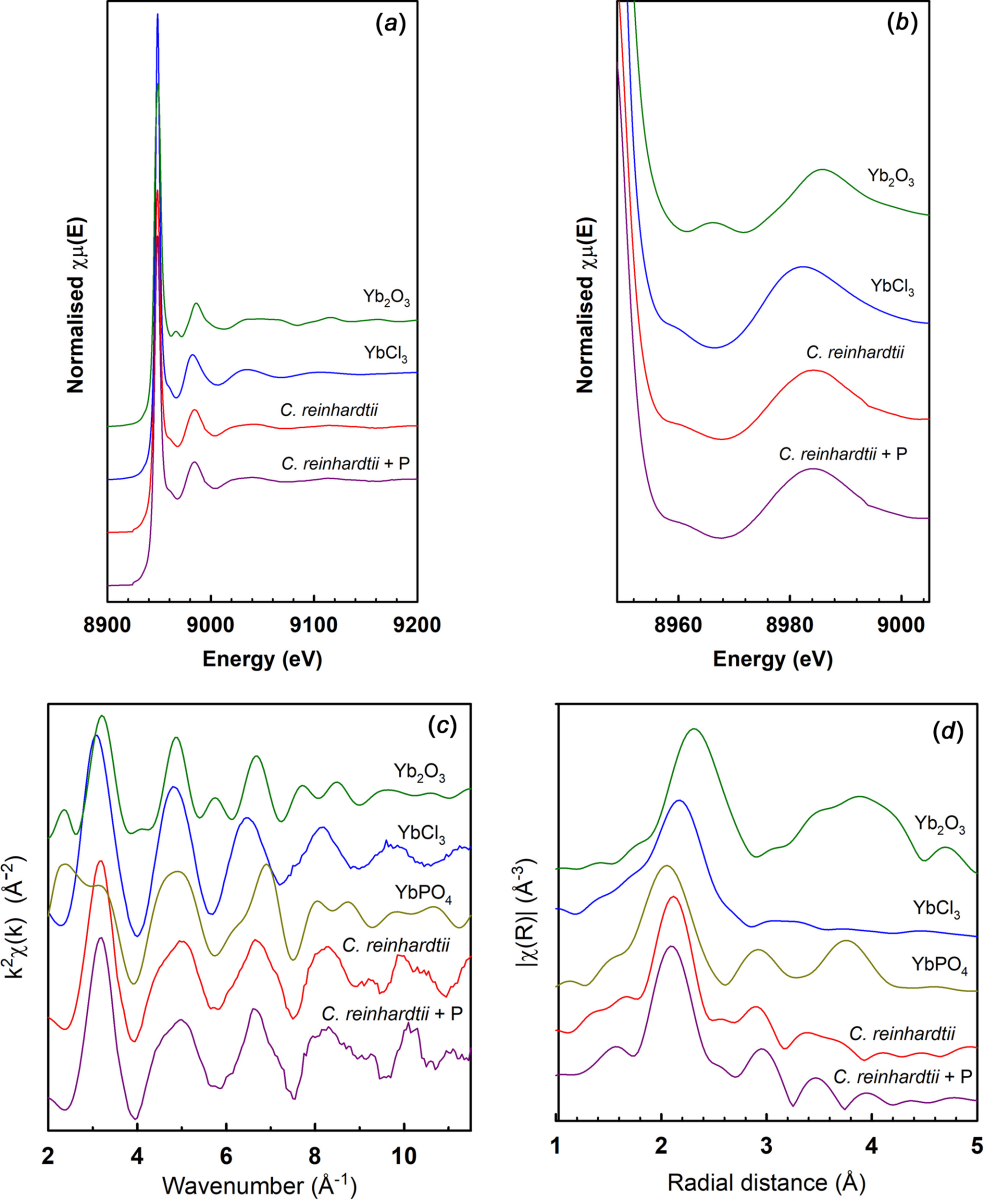
Yb L_3_-edge XAS for *C. reinhardtii* and standard com­pounds. (*a*) XANES, (*b*) near-edge region, (*c*) EXAFS function k^2^χ(k) and (*d*) Fourier transform moduli of the EXAFS spectra. k data is smoothed (11 point average). YbPO_4_ in parts (*c*) and (*d*) are derived spectra from literature crystal structures.

However, in the R plot, the differences are pronounced, where the peak at 3.0 Å in the algae samples is not present in the standards (Fig. 3 ▸). Also apparent in the R plot is the longer radial distance for the Yb—Cl bond length, forming the dominant low radial distance peak, com­pared with the Yb—O bond length in YbPO_4_ or Yb_2_O_3_. From this, the YbCl_3_ structure is easily able to be ruled out as a possible structure in *C. reinhardtii*.

The bond lengths determined from an EXAFS analysis clearly differentiate between the different reference com­pounds and identify that YbPO_4_ is the dominant form of Yb in the algae. The most intense scattering peaks determined from FEFF6 calculations from published structures for these com­pounds are given in Table 5 ▸. We were not able to get convergence of a fit of the data for Yb in *C. reinhardtii* to YbCl_3_.

The bond length of 3.0 Å corresponding to Yb—P provides evidence of the presence of YbPO_4_ in the algae samples, both with extra P and with low levels of P. To confirm this, a full fitting of the crystal structure of YbPO_4_ to the EXAFS data for the *C. reinhardtii* with P granules was performed (using *ARTEMIS* running FEFF6) (Table 5 ▸). The fit to the structure of YbPO_4_ was poor, but is probably adequate for the noisy quality of the data. The data did not require smoothing prior to fitting, because of the nature of using a Fourier transform for this fitting, which separates the high-frequency noise; however, for the purposes of displaying the experimental data, an 11-point moving average was used.

## Discussion and conclusions

Previously, we have shown that phosphate granules that form in *C. reinhardtii* are com­posed of polyphosphates, some in the form of inositol phosphate (otherwise known as phytate) (Plouviez *et al.*, 2024*a* ▸). We then showed with scanning transmission X-ray microscopy that the addition of Gd to these algae results in an association of Gd with P, including with the polyphosphate granules inside the algae (Plouviez *et al.*, 2024*b* ▸). With Ce there was an association of Ce with P but not within the algae. That previous work did not investigate the inter­actions of Yb.

P K-edge XANES on the Gd- and Ce-doped algae with extra phosphate showed some change in the chemistry of P, suggesting that there is a chemical inter­action between the rare earths and phosphate (Plouviez *et al.*, 2024*b* ▸). However, the P atom is inter­acting only at a distance, *via* the O atom bonded to phospho­rus, and therefore the chemical changes observed at the P atom are not large. A more sensitive measure of that inter­action is the work described here, where this same inter­action is observed from the rare earth atom. The change is one that is more direct, with the lanthanide inter­acting not with a chloride ion in the com­pound added, but with a phosphate ion, a larger difference in electronic inter­action. It is an inter­action specific to the lanthanide ion – so that if the phosphate is in a variety of states, with some bonded to lanthanide and some not, this will not confound the results, unlike with P K-edge XAS.

In this work, we have found that XANES was very useful in identifying the chemical structures present for Ce. There were significant differences in the near-edge spectra for the different Ce com­pounds and we had a spectrum of CePO_4_ for com­parison which provided a good match. For Gd and Yb we were not able to record spectra of the phosphate com­pounds from standards, so we had to rely on EXAFS analysis of the crystal structure. Fortunately, crystal structures of Gd and Yb phosphates are available in the literature, so it was possible to model the EXAFS spectrum from these structures to fit with the data recorded in this work and find a good match to GdPO_4_ and a reasonable match to YbPO_4_.

The L_3_-edge for Ce, Gd and Yb provided sufficient sensitivity to chemical changes to be useful for analytical purposes. Other studies have investigated this sensitivity in more detail for each of the elements at the X-ray edge (Asakura *et al.*, 2015 ▸, 2021 ▸), with many studies on Ce and some specifically on Gd (George *et al.*, 2010 ▸; Morss *et al.*, 1996 ▸; Yoon *et al.*, 2002 ▸) and Yb (Louvel *et al.*, 2015 ▸).

This study suggests that microalgae with phosphate may be a useful method of capturing rare earths from solutions. These are living organisms so the tolerance of these organisms to the presence of lanthanides also needs to be considered if this is to be a viable extraction technique. This was not directly addressed in the work presented here. However, other studies have investigated aspects of tolerance to lanthanides. In *Chlorella sp.*, Ce^3+^ slightly boosted biomass production in concentrations up to 7 µ*M*, but in *C. reinhardtii*, Ce has been found to decrease photosynthetic yield at concentrations of 1–200 µ*M* (Röhder *et al.*, 2014 ▸). Although it has also been found that in *C. reinhardtii*, Ce^3+^ does not inhibit cell growth when the Ce is stabilized by phosphate (Röhder *et al.*, 2014 ▸). The protective effect of polyphosphate accumulation has also been observed with uranium, where it increases tolerance for the cyano­bacterium *Anabaena torulosa* (Chandwadkar & Acharya, 2023 ▸).

The extraction and separation of rare earth elements (lanthanides) can be difficult due to their chemical similarities (Baldwin *et al.*, 2018 ▸). Biological processes can have very selective activity towards different elements. However, all three of the lanthanides added to the algae as chloride reacted to form phosphate com­pounds. These formed both in the presence of polyphosphate granules or when there was only a low level of P present. Nevertheless, *C. reinhardtii* or other similar microalgae may be useful in the removal of rare earths from solution, with the selectivity to different lanthanides yet to be determined.

## Figures and Tables

**Table 1:** Parameters used for data collection

Element	Region	Start energy (keV)	End energy (keV)	Step size (keV)	Step size varies by k	Time per step (*s*)	Number of points
Ce	1	5.523	5.703	0.01		1	18
(Cr ref.)	2	5.703	5.773	0.00025		1	280
	3	5.773	5.985	0.035	yes	1	134
	4	5.985	6.012	0.0002		0.2	135
	5	6.012	6.160	0.0035		1	43
							
Gd	1	7.043	7.090	0.01		1	5
(Co ref.)	2	7.090	7.131	0.00025		0.1	164
	3	7.131	7.22	0.01		1	9
	4	7.22	7.293	0.00025		1	292
	5	7.293	7.800	0.0035	yes	1	242
							
Yb	1	8.744	8.924	0.01		1	18
(Cu ref.)	2	8.924	8.994	0.00025		1	280
	3	8.994	9.493	0.0035	yes	1	240

**Table 2:** Fitting parameters used for EXAFS analysis in *ATHENA* (Ravel & Newville, 2005 ▸)

Compound		Fourier transform k_min_	Fourier transform k_max_	Fitting space	R_min_ used in fitting	R_max_ used in fitting	Longest scattering path used	Number of scattering paths used
CeO_2_		2	10	R	1	6	4.766	14
CeCl_3_·7H_2_O		2	10	R	1	6	4.939	158
CePO_4_		2	10	R	1	6	4.980	151
CePO_4_ in *C. reinhardtii*		2	10	R	1.7	5	4.980	151
Gd_2_O_3_		3	11	R	1	6	4.910	36
GdCl_3_·7H_2_O		3	11	R	1	5	2.911	2
GdPO_4_ in *C. reinhardtii*		3	11.5	R	1	6	3.227	8
Yb_2_O_3_		3	11.8	R	1.5	5	4.865	22
YbPO_4_ in *C. reinhardtii*		3	11.5	R	1.5	4.5	4.360	20

**Table 3:** Structural parameters for selected bonds in Ce com­pounds and *C. reinhardtii* calculated by *ATOMS* (Ravel & Newville, 2005 ▸) from CIF files and by fitting of the structure to the EXAFS data recorded here

Compound (CIF used)	Bond	R (Å) from *ATOMS* of CIF	Relative intensity from *ATOMS*	R (Å) from EXAFS fit	*N*	σ^2^ (Å^2^)	ΔR	*r* factor for fit of com­pound
CeO_2_	Ce—O	2.34	100	2.38	8	0.0078	−0.00057	0.15
(4343161)	Ce—Ce	3.83	67	3.82	12			
	Ce—O	4.49	51	4.48	24			
								
CeCl_3_·7H_2_O	Ce—O	2.51	100	2.51	4	0.0042	0.0058	0.13
(2201515)	Ce—O	2.54	73	2.54	3			
	Ce—Cl	2.91	42	2.92	2			
								
CePO_4_	Ce—O	2.465	100	2.46	3	0.020	−0.0092	0.11
(9001646)	Ce—O	2.53	60	2.52	2			
	Ce—O	2.58	60	2.57	2			
	Ce—O	2.64	28	2.64	1			
	Ce—O	2.78	25	2.77	1			
	Ce—P	3.20	18	3.19	1			
	Ce—P	3.28	17	3.27	1			
	Ce—P	3.75	35	3.74	3			
	Ce—Ce	4.08	30	4.07	2			
								
CePO_4_ in *C. reinhardtii*	Ce—O	2.465	100	2.47	3	0.0059	0.0063	0.14
(9001646)	Ce—O	2.53	60	2.53	2			
	Ce—O	2.58	60	2.59	2			
	Ce—O	2.64	28	2.65	1			

**Table 4:** Structural parameters for selected bonds in Gd com­pounds and *C. reinhardtii* calculated by *ATOMS* (Ravel & Newville, 2005 ▸) from CIF files and by fitting of the structure to the EXAFS data recorded here

Compound (CIF used)	Bond	R (Å) from *ATOMS* of CIF	Relative intensity from *ATOMS*	R (Å) from EXAFS fit	*N*	σ^2^ (Å^2^)	ΔR	*r* factor for fit of com­pound
Gd_2_O_3_	Gd—O	2.34	100	2.38	8	0.0078	−0.00057	0.15
(1010338)	Gd—Gd	3.83	67	3.82	12			
	Gd—O	4.49	51	4.48	24			
								
GdCl_3_·7H_2_O	Gd—O	2.51	100	2.51	4	0.0042	0.0058	0.13
(2310334)	Gd—O	2.54	73	2.54	3			
	Gd—Cl	2.91	42	2.92	2			
								
GdPO_4_ in *C. reinhardtii*	Gd—O	2.465	100	2.46	3	0.020	−0.0092	0.11
(1530459)	Gd—O	2.53	60	2.52	2			
	Gd—O	2.58	60	2.57	2			
	Gd—O	2.64	28	2.64	1			
	Gd—O	2.78	25	2.77	1			
	Gd—P	3.20	18	3.19	1			
	Gd—P	3.28	17	3.27	1			
	Gd—P	3.75	35	3.74	3			
	Gd—Gd	4.08	30	4.07	2			

**Table 5:** Structural parameters for selected bonds in Yb com­pounds and *C. reinhardtii* calculated by *ATOMS* (Ravel & Newville, 2005 ▸) from CIF files and by fitting of the structure to the EXAFS data recorded here

Compound (CIF used)	Bond	R (Å) from *ATOMS* of CIF	Relative intensity from *ATOMS*	R (Å) from EXAFS fit	*N*	σ^2^ (Å^2^)	ΔR	*r* factor for fit of com­pound
Yb_2_O_3_	Yb—O	2.36	100	2.30	6	0.011	−0.057	0.46
(1548520)	Yb—Yb	3.48	48	3.43	6			
	Yb—Yb	3.96	36	3.90	6			
	Yb—O	4.05	22	4.00	6			
	Yb—O	4.21	19	4.15	6			
								
YbCl_2_	Yb—Cl	2.793	100					
(2310334)	Yb—Cl	2.836	48					
	Yb—Cl	2.889	100					
	Yb—Cl	2.922	32					
	Yb—Yb	4.235	15					
	Yb—Cl	4.292	22					
	Yb—Yb	4.360	27					
	Yb—Yb	4.552	25					
								
YbPO_4_ in *C. reinhardtii*	Yb—O	2.27	100	2.16	4	0.0071	−0.11	0.28
(9001660)	Yb—O	2.36	92	2.24	4			
	Yb—P	2.98	29	2.87	2			
	Yb—O—P	3.43	10	3.32	8			
	Yb—O—O	3.69	42	3.58	8			
	Yb—Yb	3.72	56	3.60	4			

## Data Availability

Raw data and fitted data are available on request.
